# A Tale of Two Pandemics: Antimicrobial Resistance Patterns of *Enterococcus* spp. in COVID-19 Era

**DOI:** 10.3390/antibiotics12020312

**Published:** 2023-02-02

**Authors:** Dan Alexandru Toc, Alexandru Botan, Ana Maria Cristia Botescu, Vlad Dumitru Brata, Ioana Alina Colosi, Carmen Costache, Lia Monica Junie

**Affiliations:** 1 Department of Microbiology, Iuliu Hatieganu University of Medicine and Pharmacy, 8 Victor Babeș Street, 400000 Cluj-Napoca, Romania; 2 Faculty of Medicine, Iuliu Hatieganu University of Medicine and Pharmacy, 8 Victor Babeș Street, 400000 Cluj-Napoca, Romania

**Keywords:** *Enterococcus*, COVID-19, antimicrobial resistance, pandemic

## Abstract

Although the COVID-19 pandemic has held the spotlight over the past years, the antimicrobial resistance (AMR) phenomenon continues to develop in an alarming manner. The lack of strict antibiotic regulation added to the overuse of antimicrobials fueled the AMR pandemic. This paper aims to analyze and identify the impact of the COVID-19 pandemic on antibiotic resistance patterns of *Enterococcus* spp. The study was designed as a retrospective observational study. *Enterococcus* spp. infections data were collected from one academic hospital in Cluj-Napoca, Romania over 18 months. A statistical analysis was performed to compare antibiotic resistance phenotypes identified. We recorded an increase in the isolation rates of *Enterococcus* spp. strains, from 26 isolates (26.53%) during Period A (November 2020–April 2021) to 42 strains (42.85%) during Period C (November 2021–April 2022). The number of strains with resistance to vancomycin increased from 8 during Period A to 17 during Period C. Of the total 36 strains with resistance to vancomycin, 25 were identified as *E. faecium*. SARS-CoV-2 patients (*n* = 29) proved to be at risk to develop an *E. faecium* co-infection (*n* = 18). We observed that strains with resistance to ampicillin (*n* = 20) and vancomycin (*n* = 15) are more often isolated from these patients. All changes identified in our study are to be considered in the light of COVID-19 pandemic, highlighting the threatening AMR phenomenon in Romania. Further studies should be performed to quantify the worldwide effects of these pandemics.

## 1. Introduction

Antimicrobials have proven to be an effective tool in the fight against bacteria and fungi over the years, becoming nearly indispensable for medical practice all-over the world. Antibiotics are an essential tool in treating human infections, especially in developing countries which may lack awareness when it comes to hygienization [1]. Unfortunately, due to the ability of the microorganisms to develop resistance to antimicrobial substances, these therapies became less and less effective in time. This phenomenon of antimicrobial resistance (AMR) was linked with the wide availability and the overuse of antimicrobial substances [2]. Moreover, the lack of proper epidemiological surveillance (especially in healthcare units) makes it impossible for specialists to develop customized protocols for high-efficiency low-risk antibiotic therapies [3]. To prevent the misuse and the further development of this AMR phenomenon, it became of utmost importance that both the commercialization and prescription of antibiotics be highly regulated [4].

Worldwide, a link was identified between the use of antibiotics and the knowledge about antimicrobial resistance. It appears that the lack of education on appropriate usage of antibiotics is strongly connected to the spread of AMR. Unfortunately, surveys on antibiotic usage show not just the level of misinformation, but also the increasing number of people targeted by this phenomenon [5].

The difference between national systems of regulation and surveillance of antibiotics use can be seen in the demographic distribution of AMR patterns. When it comes to Europe, a trend for more resistant bacteria was identified in the eastern region. Strains of *Klebsiella pneumoniae* resistant to third-generation cephalosporines and *Acinetobacter* spp. carbapenem-resistant bacteria were found more frequently in southern and eastern parts of the European Region. While these strains respected a demographic distribution, there are multi-drug resistant (MDR) bacteria that are widely spread in the European region such as methicillin-resistant *Staphylococcus aureus* (MRSA) or vancomycin-resistant *Enterococcus faecium* (VRE) [6].

Although the entire eastern region of Europe was affected by the AMR problem, Romania has represented a point of interest in the past years. In comparison with other countries from European Union (EU)/European Economic Area (EEA), Romania reported an increasing number of MDR bacteria which seemed concerning. The main problem identified in Romania was the lack of awareness when it comes to surveillance of healthcare-associated infections (HAIs) and antibiotic consumption on a local level. Moreover, the absence of these measures made it impossible for healthcare providers to elaborate efficient treatment guidelines in order to achieve a vigilant use of antimicrobials [7].

Intensive care units (ICUs) proved to be the most affected by the AMR phenomenon. There were previously described errors in the administration of antibiotic prophylaxis and empirical treatments which played a major role in the MDR healthcare-associated infections (HAIs). The most worrisome part is the shortage of research to address this problem, especially in the past years in which the attention was shifted to the COVID-19 pandemic [8,9].

During the COVID-19 pandemic, antibiotic consumption increased due to the absence of proper antiviral therapies and efficient guidelines. Moreover, fungal and bacterial co-infections in SARS-CoV-2 patients became new challenges for healthcare systems all-over the world [10,11,12,13]. In most cases, antibiotic therapies were inappropriately prescribed, there being no evidence of any co-infection. Moreover, most countries reported an increase in self-medication with antibiotics. This phenomenon amplified the pre-existing AMR problem, especially in critically ill patients, which led to an increased mortality rate in these patients [14,15,16].

Pathogens causing nosocomial infections, due to their prolonged contact with antimicrobials, represent the main targets of the AMR pandemic. Even before the COVID-19 era, the ESKAPE group (*Enterococcus faecium*, *Staphylococcus aureus*, *Klebsiella pneumoniae*, *Acinetobacter baumannii*, *Pseudomonas aeruginosa* and *Enterobacter* spp.) was repeatedly incriminated in human infections with MDR bacterial strains [14,17]. Extended Spectrum Beta-Lactamase (ESBL), Carbapenem Resistant Enterobacterales (CRE), MRSA, and VRE strains are responsible for the highest mortality rates in ICUs [8,18,19].

A worrisome synergy was noticed between the SARS-CoV-2 virus and *Enterococcus* spp. It appears that SARS-CoV-2 pneumonia alters the intestinal microbiota, allowing *Enterococcus* strains to proliferate and increasing intestinal permeability. These changes can explain the increased number of patients suffering from COVID-19 and associating an *Enterococcus* spp. bloodstream infection (BSI) [20].

As previously presented, the first step in fighting this AMR pandemic is represented by a strict local/regional epidemiological surveillance that allows specialists to provide customized therapies for these HAIs. The lack of epidemiological reports in Romania became a financial burden for healthcare systems in the country, due to the increasing number of nosocomial infections with MDR bacteria. This study aims to provide data regarding invasive infections with *Enterococcus* spp. in a single academic hospital in Cluj-Napoca, Romania, both in patients with and without a SARS-CoV-2 infection. Moreover, this paper seeks to identify the effects of the COVID-19 pandemic over the antimicrobial resistance patterns of *Enterococcus* spp. to encourage further studies and the development of local guidelines of treatment.

## 2. Results

### 2.1. Analysis of Enterococcus spp. Infections

A number of 98 patients were confirmed with an *Enterococcus* spp. infection during the time of our study (1 November 2020–30 April 2022). *E. faecalis* was the pathogen incriminated in 54 infections, *E. faecium* was identified in 42 samples, and 2 infections were confirmed with *E. gallinarum*. Of these patients, 29 were confirmed with a SARS-CoV-2 infection. Distribution of *E. faecalis* and *E. faecium* among these patients is presented in Table 1.

Among the collected samples, *E. faecalis* was mostly isolated from wound drainage samples, while *E. faecium* was mostly reported in blood samples. A summary of the collected samples is presented in Table 2.

The detected phenotypes, based on their minimum inhibitory concentrations (MICs), on VITEK-2 antibiotic susceptibility testing (AST) are presented in Table 3. The most susceptible strains were *E. faecalis* strains, while *E. faecium* harbored most *vanA* like (MIC for Vancomycin >64 mg/L, MIC for Teicoplanin >16 mg/L) and *vanB* like (MIC for Vancomycin >4 mg/L, MIC for Teicoplanin >0.5 mg/L) phenotypes. All *E. gallinarum* presented *vanC* like (low level resistance to Vancomycin) phenotype.

### 2.2. Analysis of COVID-19 Co-Infection

Data regarding gender distribution, survivability, and length of stay in the COVID-19 group are presented in Table 4. During this study, 29 patients admitted in our unit with a SARS-CoV-2 pneumonia developed an *Enterococcus* spp. co-infection. Out of these patients, 13 were females and 16 were males. Only 17 of 29 patients were deceased.

### 2.3. Analysis of Enterococcus spp. Infection over Time

The number of confirmed infections with *Enterococcus* spp. increased during the periods of this study. *E. faecalis* was predominantly isolated during Period B (1 May 2021 to 30 October 2021) while the number of infections with *E. faecium* increased during Period C (1 November 2021 to 30 April 2022). All *E. gallinarum* strains were isolated during Period C. More information regarding the distribution during the periods of this study is available in Table 5 and Table 6.

The results of AST for each period of our study are presented in Table 7. Statistically significant differences were reported for streptomycin (STR), linezolid (LNZ), and tetracycline (TET).

**Figure 1:**
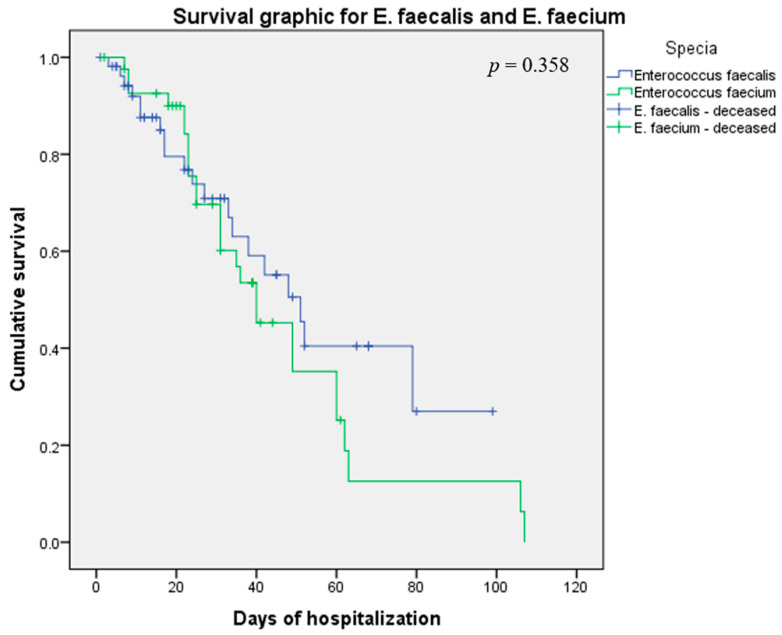
Kaplan Meier survival analysis for *E. faecalis* and *E. faecium*.

Survival rates of patients infected with *E. faecalis* and *E. faecium* are presented in Figure 1. No statistically significant difference was identified between the 2 groups.

**Figure 2:**
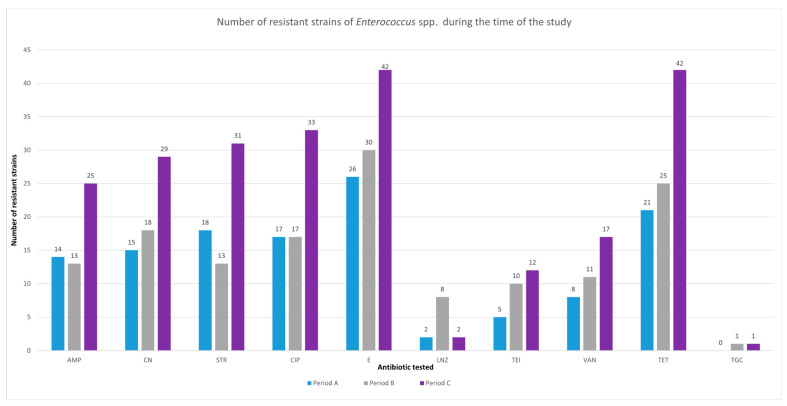
Distribution of *Enterococcus* spp. resistant strains over the period of this study. Abbreviations: AMP, ampicillin; CN, gentamicin; STR, streptomycin; CIP, ciprofloxacin; E, erythromycin; LNZ, linezolid; TEI, teicoplanin; VAN, vancomycin; TET, tetracycline; TGC, tigecycline.

The number of resistant *Enterococcus* spp. strains are presented in Figure 2 and Table 8. There is no resistance to Tigecycline (TGC) reported during Period A. Strains of *E. faecalis* with resistance to TGC are reported during period B and C. An increase in the number of resistant strains was reported for most tested antibiotics when Period A was compared to Period C.

### 2.4. Analysis of Antibiotic Resistance Patterns

Ampicillin, streptomycin, ciprofloxacin, teicoplanin, and vancomycin resistance was more likely to occur in strains of *E. faecium*, rather than *E. faecalis*. The odds ratio for these associations are presented in Table 9.

COVID-19 co-infection occurred more often in patients infected with *Enterococcus* spp. strains with resistance to ampicillin and vancomycin. The results for all tested antibiotics are presented in Table 10.

Regarding survivability, death was more likely to occur in patients infected with *Enterococcus* spp. strains with resistance to ciprofloxacin. Results of the Chi-square/ Fisher’s test and the Odds ratios associated with them are presented in Table 11.

In infections produced by VRE, Linezolid and Tigecycline are two last resort antibiotics. Table 12 presents the MICs distribution for all the *Enterococcus* spp. isolates included in our study.

## 3. Discussion

### 3.1. Enterococcus spp. during the COVID-19 Pandemic

Although the COVID-19 pandemic seems to have come to an end, its impact on the HAIs with MDR pathogens is still up for debate. Several studies reported a rise in antibiotic use, but the aftermath of this increase remains unclear [21,22,23]. Previous viral pandemics taught us that a secondary bacterial or fungal infection provides a poor outcome for the patient. Although there have been several mechanisms described that aimed to explain the relationship between viruses and bacteria regarding the infection pathogenesis, the SARS-CoV-2 involvement remains understudied. The existing data show that this virus seems to rely on regulating the gene expressions in monocytes. Furthermore, some studies show a gastrointestinal involvement of SARS-CoV-2. Considering the microbiome diversity at this situs, a possible virus-induced immunosuppression may lead to secondary bacterial infection. Thus, a specific guideline should be implemented for the use of antibacterial and antifungal drugs in SARS-CoV-2 infections [24].

The goal of our study was to describe the changes in antimicrobial resistance patterns in *Enterococcus* spp. during the COVID-19 pandemic and to analyze the relationship between these two microorganisms.

During the 18 months of our study, there an increase in the number of invasive infections with *Enterococcus* spp. was noticed. Although there was no statistically significant difference between the three time periods (*p* > 0.05), we noticed an increased number of infections with *Enterococcus* spp. (42.85%) in the last 6 months of our study (November 2021 to April 2022). The increasing number of *Enterococcus* spp. infections was described also by M. Polemis et al. in samples from ICUs in Greece [25]. Following the findings of their study, we noticed in our center that the majority of infections were caused by *E. faecium,* most of them being bloodstream infections (BSIs).

Regarding gender distribution, *E. faecium* and *E. faecalis* infections were evenly distributed among male and female patients. Although, there was no statistically significant difference between gender distribution (*p* > 0.05), a trend was noticed for *E. faecalis* infections affecting mainly the male patients.

In patients suffering from SARS-CoV-2 pneumonia, we noticed that an infection with *E. faecium* was more likely to occur (*p* < 0.05) when compared to the *E. faecalis* group. Although, no significant difference in mortality for patients with SARS-CoV-2 co-infection was found, infections with *E. faecium* had significant lower rates of survivability (*p* < 0.05) in comparison with *E. faecalis* and *E. gallinarum*. These results suggest that the severity of these infections was not the presence of SARS-CoV-2 co-infections, but the *Enterococcus* spp. strain that infected the patient. However, the number of *E. galinarum* strains was low and therefore the real impact of this species remains to be established. In the light of these findings, better management of the *E. faecium* infections could have provided better outcomes for these patients.

The site of infections proved to be of great importance as well. Although isolated from samples as puss (*n* = 2), cerebrospinal fluid (*n* = 1), or peritoneal fluid (*n* = 3), *E. faecium* was isolated significantly more often (*p* < 0.05) from blood samples (*n* = 21). Although our small number of strains did not allow us to perform an in-depth analysis, different authors described BSIs with *E. faecium* in patients with SARS-CoV-2, especially in ICUs. The alterations that COVID-19 brings to the gut microbiota may be the main culprit of the high prevalence of *Enterococcus* spp. BSI [20,26,27]. *E. faecium* seems more likely to cross the intestinal barrier when compared to *E. faecalis*. Moreover, all central line bacteriemia (*n* = 4) in our study were caused by *E. faecium*, findings previously described also by S. Hughes et al. [28]. Furthermore, central venous catheter (CVC) infections were previously associated with the ability of *Enterococcus* spp. to form biofilms. The biofilms formed by MDR bacterial strains may impact the prognosis of patients with CVC infections [29,30,31,32,33].

On the other hand, *E. faecalis* presented a more heterogenous distribution, being isolate most frequently from wound samples (*n* = 25) and urine samples (*n* = 4). We consider that further studies may be needed in order to better understand how the site of infections can impact the treatment plan for these patients.

When it comes to antibiotic therapies, vancomycin remains one of the used antimicrobials in *Enterococcus* spp. infections. This phenomenon represents a risk factor for increasing resistance of *Enterococcus* spp. strains to vancomycin. The vancomycin resistant Enterococci (VRE) were repeatedly reported in literature to burden the healthcare systems all-over the world, causing invasive infections resistant to usual treatment protocols. In our study, we identified that *E. faecium* strains had significantly higher rates (*p* < 0.05) to harbor a *vanA* like phenotype in comparison with *E. faecalis*. In association with the lower *E. faecium* survivability rates, we can consider that *vanA* resistance phenotype represents a factor of bad prognosis in patients with an *E. faecium* infection [34].

The results of our Kaplan Meier survival analysis, although not statistically significant (*p* > 0.05), provides useful data regarding the clinical impact of *Enterococcus* spp. infections. While *E. faecalis* infections are associated with lower survivability rates in the first 20 to 40 days of hospitalization, *E. faecalis* infections survivability rates dropped after this time period. Though there is no significant difference in the average of the hospitalization days (*p* > 0.05), *E. faecium* patients spent an average of 5.5 more days in our hospital than *E. faecalis* patients. The longer length of stay and lower survivability rates can be linked with the resistance of these strains to vancomycin and other usual antibiotics, lengthening the time needed to treat these infections. D. R. Giacobbe et al. described the role of enterococcal BSI in the clinical evolution of COVID-19 patients [35]. Their study reported an increased incidence of *Enterococcus* spp. BSI in critically ill COVID-19 patients and higher mortality rates for these patients.

As previously discussed, unregulated antibiotic use seems to evolve and its impact on the increased number of highly resistant strains of bacteria becomes relevant. In the light of COVID-19 pandemic aftermath analysis, we can assess the involvement of previous antibiotic use in developing resistant strains of bacteria that are able to promote infections. Considering the struggle of the silent AMR pandemic, it does not come as a surprise that *Enterococcus* spp. emerged as a real threat to healthcare systems all around the world. The association of COVID-19 and infections produced by *Enterococcus* spp. has been evaluated in several articles that showcase an increase in the total length of hospitalization. Since VRE is constantly under surveillance by healthcare professionals, several protocols have been proposed in order to properly tackle the issue of VRE spread. However, due to the increased pressure on the healthcare system that was imposed by the COVID-19 pandemic, several breaches that led to hospital spread of VRE have been described in literature. To provide a better supervision of these strains, further studies that aim to develop better guidelines are required [36,37,38,39,40,41,42].

### 3.2. Over-Time Analysis of Enterococcus spp. Infections

The overall results of our study provide us with data to interpret the impact of *Enterococcus* spp. infections during the 18 months of our study, in association with the pressure COVID-19 put on our healthcare system. In order to identify the aftermath of the SARS-CoV-2 pandemic, we needed an over-time analysis of these results. We evenly divided our study into three periods of 6 months each representing the first cold season COVID-19 pandemic (Period A), the warm season COVID-19 pandemic (Period B), and the second cold season and the aftermaths (Period C). Although not statistically significant (*p* > 0.05), both cold seasons of our study registered more COVID-19 positive patients (*n* = 8 for Period A and *n* = 16 for period C) than Period B (*n* = 5). These findings can be the result of both weather conditions and national restrictions used to lower the number of SARS-CoV-2 cases.

While the number of *Enterococcus* spp. infection increased and we noticed significantly more VRE *E. faecium* with lower survivability rates, we continued our analysis in order to identify if these findings represent the results of the management of COVID-19 pandemic. The results of our antibiotic susceptibility tests (ASTs) provided useful data if analyzed over-time. Most of the tested antibiotics reported increased resistance during Period C in comparison with Period A. Strains resistant to gentamicin, teicoplanin and vancomycin were isolated more frequently as the time passed. The increasing resistance to these commonly used antibiotics can be the result of misuse of antimicrobial therapies in patients with SARS-CoV-2 pneumonia. Although the antibiotic therapies were reported to have no beneficial effect in COVID-19 infection, it appears that their increased use selected more resistant bacterial strains incriminated in nosocomial infections.

An unusual resistance pattern was identified for linezolid resistant *Enterococcus* spp. strains. During our study, the resistance to linezolid reached a peak during Period B and decreased during period C. In the light of these increasing resistance patterns, we shift our attention to linezolid to provide a solution to MDR *Enterococcus* strains. Resistance to linezolid is defined as an MIC greater than 4 mg/L according to the EUCAST 2023 guideline (available on the EUCAST website: https://www.eucast.org [accessed on 22 January 2023]). In our study, 12 strains are reported as resistant. Due to the retrospective nature of this study, we were unable to perform molecular analysis in order to characterize the resistance mechanism.

Lowest resistance rates were reported for tigecycline (TGC). During Period A, no strains of *Enterococcus* spp. with resistance to TGC were identified, but resistance to TGC was identified during Period B (*n* = 2) and Period C (*n* = 2). Unusual for our study is that all TGC resistant strains were identified as *E. faecalis*. Although, the number of resistant strains were too low to provide statistical significance (*p* > 0.05), we consider this trend worth supervising. Resistance to tigecycline is defined as an MIC greater than 0.25 mg/L according to the EUCAST 2023 guideline (available on the EUCAST website: https://www.eucast.org [accessed on 22 January 2023]). Molecular analysis to identify the resistance mechanism was unavailable.

In our center, *E. faecium* proved to be a risk factor for harboring resistance to ampicillin (*p* < 0.01), streptomycin (*p* < 0.05), ciprofloxacin (*p* < 0.01), teicoplanin (*p* < 0.01), and vancomycin (*p* < 0.01). These findings highlight the importance of quick and efficient bacterial identification for *Enterococcus* spp. strains as there are differences when it comes to the resistance patterns of *E. faecium* and *E faecalis*. Corroborating these results with the increasing number of resistant strains during our study, we consider that *E. faecalis* strains present a higher susceptibility to acquire antibiotic resistance genes, representing a bigger threat for the healthcare systems in our region.

In COVID-19 patients, we identified statistically significant differences for Ampicillin (*p* < 0.05) and Vancomycin (*p* < 0.05). The presence of SARS-CoV-2 infection proved to be associated with higher chances of selecting an *Enterococcus* strain with resistance to ampicillin (OR = 2.569) and vancomycin (OR = 2.707).

The survivability rates in association with antibiotic resistance were not statistically significant different for most of the antibiotics tested. We registered a significant higher mortality rate for strains with resistance to ciprofloxacin (*p* < 0.05, OR = 3.015). This result can serve as a warning sign for medical professionals when it comes to using ciprofloxacin as a treatment alternative for patients at high-risk. Y. B. Kim et al. described *Enterococcus* spp. strains with resistance to ciprofloxacin isolated from chicken meat [43]. It is important highlights the possibility of contamination through food products and raise awareness when it comes to this understudied field.

### 3.3. Enterococcus gallinarum and the Emergence of Other Enterococci

Other Enterococci (OE) were reported in literature more frequently in the past few years [44]. As for our study, we cannot identify strains of OE during the first 12 months. A surprising discovery was the emergence of *E. gallinarum* infections (*n* = 2) during Period C. We identified no association between *E. gallinarum* with SARS-CoV-2 patients, and the survivability rates for patients infected with these strains were 100%. It is important to consider that both *E. gallinarum* strains were isolated from cerebrospinal fluid (CSF). During the 18 months of our study, we isolated only 4 strains on *Enterococcus* spp. from CSF, and 50% of these strains were represented by *E. gallinarum*. Although, the number of strains is insufficient to provide significance, we must pay attention to this trend of increasing *E. gallinarum* infections in neurological pathologies [45,46].

### 3.4. COVID-19, Enterococccus spp. and the Use of Probiotics

It is known that one of the first steps in infection pathogenesis is the ability of the bacterial strains to colonize different surfaces and tissues. From here, every disruption of the surface integrity provides the premises for the bacterial strain to emerge as etiologic agent for the infection. This basic model to infection applies to *Enterococcus* spp. as well, considering the wide variety of factors that are present. Recent studies show that hospital adapted linages of Enterococci are able to colonize the human gut developing a reservoir from where these strains can cross the gut barrier and produce systemic infections. Translocation of Enterococci from the gut into the bloodstream is enhanced by several factors like the extensive use of antibiotics which challenge the microbiome balance as well as inflammation. Thus, the extensive use of antimicrobials during the COVID-19 pandemic along with the systemic inflammation that the SARS-CoV-2 virus produces represents the arguments for the increased number of *Enterococcus* spp. infections in COVID-19 patients.

Recent studies show that commensal *E. faecalis* is able to destroy the VRE strain V583. This is achieved to the production of a specific pheromone peptide called cOBI. This shows that in time VRE colonization can be overcome by providing a balanced intestinal microbiome. It becomes clear that tackling dysbiosis represents an acceptable therapeutic target in patients with SARS-CoV-2 pneumonia since it can provide immune homeostasis in the gut. Choosing the right probiotic remains an issue, further studies being required in order to address the pros and cons of each valid candidate [47,48,49].

### 3.5. Limitations and Implications

Regarding the limitations of our study, one of the first things worth mentioning is the retrospective nature of this paper. Furthermore, it is important to mention that all the data included are from only one academic hospital in Romania. In addition, only a small number of samples met our inclusion criteria and therefore it is difficult to estimate the impact of our findings on a larger scale. Moreover, our facility provides bacterial identification only using the VITEK-2 system. A more accurate diagnostic tool was not available.

Our study was designed to highlight the danger that AMR phenomenon represents to our healthcare system and our results confirmed this emerging threat. Moreover, we identified changes in the pattern of antimicrobial resistance of *Enterococcus* spp. strains that may be linked to the on-going COVID-19 pandemic. Further studies should consider a multicentric approach, in order to validate our results on a larger scale.

## 4. Materials and Methods

This study was designed as an observational, retrospective study, including data that were gathered from a single academic hospital in Cluj-Napoca, Romania. We collected data regarding *Enterococcus* spp. infections during the COVID-19 pandemic period from 1 November 2020 to 30 April 2022.

The data included in this study were from patients admitted in our unit with at least one episode of infection with *Enterococcus* spp. To be included in this study, (1) the infection had to be confirmed by a bacterial identification on VITEK-2 Compact (bioMérieux, Inc., Marcy l’Etoile, France) and (2) an antibiotic susceptibility test should have been performed on VITEK-2 Compact (bioMérieux, Inc., Marcy l’Etoile, France).

Ninety-eight patients fulfilled the inclusion criteria. Medical records of these patients were reviewed, and the following data have been anonymously stored in our database: gender, date of admission, SARS-CoV-2 infection status, length of stay, survival, sample analyzed, *Enterococcus* spp. species identified and the results of the susceptibility tests for Ampicillin, Gentamicin, Streptomycin, Ciprofloxacin, Erythromycin, Linezolid, Teicoplanin, Vancomycin, Tetracycline and Tigecycline. We divided the timeline of our study (18 months) into 3 evenly distributed time periods: Period A from 1 November 2020 to 30 April 2021; Period B from 1 May 2021 to 30 October 2021 and Period C from 1 November 2021 to 30 April 2022.

### 4.1. Microbiological Assesment

All samples were processed according to EUCAST guidelines. The bacterial identification was performed using isolated colonies via VITEK-2 Compact using the VITEK^®^ 2 GP ID card (bioMérieux, Inc., Marcy l’Etoile, France). The antibiotic susceptibility testing was performed using the VITEK^®^ 2 AST-P592 card (bioMérieux, Inc., Marcy l’Etoile, France).

### 4.2. Statistical Assesment

The data we collected were stored in a Microsoft Excel database. For our ordinal data, a Shapiro-Wilk Test for Normality was performed. Nominal data groups were compared using the Chi-square test or Fisher’s test (based on sample size). Means comparison was performed using One-Way ANOVA and Student test. Log-rank analysis and Cox regression were performed for the survival analysis. We considered *p*-value statistically significant for values less than 0.05. The entire statistical analysis was performed using the IBM^®^ SPSS^®^ Statistics 20.

## Figures and Tables

**Table 1:** Analysis of gender, SARS-CoV-2 infection, survivability, and length of stay among patients with *E. faecalis* and *E. faecium*.

	*E. faecalis*	*E. faecium*	*E. gallinarum*	*p*
Gender				
Female	25	21	0	0.561
Male	29	21	2	
SARS-CoV-2				
Positive	11	18	0	0.037
Negative	43	24	2	
Survivability				
Survived	34	16	2	0.015
Deceased	20	26	0	
Length of stay				
Average hospitalization days	29.07	34.62	1	0.093

**Table 2:** Distribution of *E. faecalis*, *E. faecium* and *E. gallinarum* among the samples collected from patients.

	*E. faecalis*	*E. faecium*	*E. gallinarum*	*p*
Blood	16	21	0	0.022
Wound drainage	25	10	0	
Other samples	13	11	2	
Urine	4	0	0	
Cerebrospinal fluid	1	1	2	
Central venous catheter	0	4	0	
Puss	1	2	0	
Puncture fluid	1	0	0	
Peritoneal fluid	3	3	0	
Pleural fluid	1	0	0	
Bronchoalveolar lavage (BAL)	0	1	0	
Tracheal aspirate	2	0	0	

**Table 3:** Distribution of antibiotic resistance phenotypes of *E. faecalis*, *E. faecium*, and *E. gallinarum* on VITEK-2 antibiotic susceptibility testing (AST).

	Susceptible	*vanA* Like	*vanB* Like	*vanC* Like	*p*
*E. faecalis*	45	7	2	0	<0.01
*E. faecium*	17	20	5	0	
*E. gallinarum*	0	0	0	2	

**Table 4:** Distribution of gender, survivability, and length of stay among patients with SARS-CoV-2 pneumonia and *Enterococcus* spp. co-infection.

	COVID-19 Positive	COVID-19 Negative	*p*
Gender			
Female	13	33	0.481
Male	16	36	
Survivability			
Survived	12	40	0.183
Deceased	17	29	
Length of stay			
Average hospitalization days	33.69	29.70	0.440

**Table 5:** Number of infections with *Enterococcus* spp. during the time of this study.

	*E. faecalis*	*E. faecium*	*E. gallinarum*	Total (%)	*p*
Period A	16	10	0	26 (26.53%)	0.181
Period B	20	10	0	30 (30.61%)	
Period C	18	22	2	42 (42.85%)	

Period A (from 01 November 2020 to 30 April 2021); Period B (from 01 May 2021 to 30 October 2021); Period C (from 01 November 2021 to 30 April 2022).

**Table 6:** Number of co-infections with *Enterococcus* spp. and SARS-CoV-2 during this study.

	*COVID +*	*COVID −*	*p*
Period A	8	18	0.163
Period B	5	25	
Period C	16	26	

Period A (from 01 November 2020 to 30 April 2021); Period B (from 01 May 2021 to 30 October 2021); Period C (from 01 November 2021 to 30 April 2022).

**Table 7:** Results of antibiotic susceptibility testing (AST) for all strains of *Enterococcus* spp. during the 18 months of the study.

	Period A	Period B	Period C	*p*
Ampicillin				
R	14	13	25	0.425
S	12	17	17	
Gentamicin				
R	15	18	29	0.598
S	11	12	13	
Streptomycin				
R	18	13	31	0.023
S	8	17	11	
Ciprofloxacin				
R	17	17	33	0.141
S	9	13	9	
Erythromycin				
R	26	30	42	-
S	0	0	0	
Linezolid				
R	2	8	2	0.018
S	24	22	40	
Teicoplanin				
R	5	10	12	0.528
S	21	20	30	
Vancomycin				
R	8	11	17	0.719
S	18	19	25	
Tetracycline				
R	21	25	42	0.004
S	5	5	0	
Tigecycline				
R	0	1	1	0.942
S	26	29	41	

Abbreviations: R, resistant: S, susceptible.

**Table 8:** Distribution of resistant strains of *E. faecalis*, *E. faecium*, and *E. gallinarum* during the periods of this study.

		AMP	CN	STR	CIP	E	LNZ	TEI	VAN	TET	TGC	Total Number of Strains
Period A	*E. faecalis*	4	7	10	7	16	2	2	3	14	0	16
	*E. faecium*	10	8	8	10	10	0	3	5	7	0	10
	*E. gallinarum*	-	-	-	-	-	-	-	-	-	-	0
Period B	*E. faecalis*	4	12	7	9	20	6	4	5	18	1	20
	*E. faecium*	9	6	6	8	10	2	6	6	7	0	10
	*E. gallinarum*	-	-	-	-	-	-	-	-	-	-	0
Period C	*E. faecalis*	4	12	12	12	18	1	1	1	18	1	18
	*E. faecium*	21	17	19	21	22	1	11	14	22	0	22
	*E. gallinarum*	0	0	0	0	2	0	0	2	2	0	2

Abbreviations: AMP, ampicillin; CN, gentamicin; STR, streptomycin; CIP, ciprofloxacin; E, erythromycin; LNZ, linezolid; TEI, teicoplanin; VAN, vancomycin; TET, tetracycline; TGC, tigecycline.

**Table 9:** Statistical analysis of antibiotic resistant strains in *E. faecalis* and *E. faecium* groups.

Antibiotic	R	S	*p*	OR
Ampicillin				
*E. faecalis*	12	42	<0.01	0.014
*E. faecium*	40	2		
Gentamicin				
*E. faecalis*	31	23	0.132	0.478
*E. faecium*	31	11		
Streptomycin				
*E. faecalis*	29	25	0.017	0.316
*E. faecium*	33	9		
Ciprofloxacin				
*E. faecalis*	28	26	<0.01	0.083
*E. faecium*	39	3		
Linezolid				
*E. faecalis*	9	45	0.162	2.600
*E. faecium*	3	39		
Teicoplanin				
*E. faecalis*	7	47	<0.01	0.164
*E. faecium*	20	22		
Vancomycin				
*E. faecalis*	9	45	<0.01	0.136
*E. faecium*	25	17		
Tetracycline				
*E. faecalis*	50	4	0.325	2.083
*E. faecium*	36	6		
Tigecycline				
*E. faecalis*	2	52	0.109	0.000
*E. faecium*	0	42		

Abbreviations: R, resistant; S, susceptible; OR, Odds Ratio.

**Table 10:** Statistical analysis of antibiotic resistant strains among COVID-19 patients.

Antibiotic	R	S	*p*	OR
Ampicillin				
COVID-19 positive	20	9	0.048	2.569
COVID-19 negative	32	37		
Gentamicin				
COVID-19 positive	19	10	0.822	1.149
COVID-19 negative	43	26		
Streptomycin				
COVID-19 positive	20	9	0.645	1.323
COVID-19 negative	42	25		
Ciprofloxacin				
COVID-19 positive	24	5	0.058	2.902
COVID-19 negative	43	26		
Linezolid				
COVID-19 positive	4	25	1.000	1.180
COVID-19 negative	8	59		
Teicoplanin				
COVID-19 positive	12	17	0.053	2.541
COVID-19 negative	15	54		
Vancomycin				
COVID-19 positive	15	14	0.037	2.707
COVID-19 negative	19	48		
Tetracycline				
COVID-19 positive	27	2	0.508	1.831
COVID-19 negative	59	8		
Tigecycline				
COVID-19 positive	0	29	0.354	0.000
COVID-19 negative	2	67		

Abbreviations: R, resistant; S, susceptible; OR, Odds Ratio.

**Table 11:** Statistical analysis of the impact of antibiotic resistance on survivability.

Antibiotic	Deceased	Survived	*p*	OR
Ampicillin				
R	28	24	0.161	1.815
S	18	28		
Gentamicin				
R	30	32	0.834	1.172
S	16	20		
Streptomycin				
R	31	31	0.530	1.400
S	15	21		
Ciprofloxacin				
R	37	30	0.018	3.015
S	9	22		
Linezolid				
R	6	6	1.000	1.150
S	40	46		
Teicoplanin				
R	16	11	0.175	1.988
S	30	41		
Vancomycin				
R	19	17	0.408	1.449
S	27	35		
Tetracycline				
R	42	46	0.746	1.370
S	4	6		
Tigecycline				
R	0	2	0.101	0.000
S	46	50		

Abbreviations: R, resistant; S, susceptible; OR, Odds Ratio.

**Table 12:** Minimum inhibitory concentrations (MICs) for Linezolid and Tigecycline.

Linezolid		Tigecycline	
MIC (mg/L)	Number of Isolates	MIC (mg/L)	Number of Isolates
1	24	≤0.12	96
2	62	0.5	1
≥8	12	1	1
Total	98	Total	98

## Data Availability

Not applicable.
